# Is Pregnancy Associated with Severe Dengue? A Review of Data from the Rio de Janeiro Surveillance Information System

**DOI:** 10.1371/journal.pntd.0002217

**Published:** 2013-05-09

**Authors:** Carolina Romero Machado, Elizabeth Stankiewicz Machado, Roger Denis Rohloff, Marina Azevedo, Dayse Pereira Campos, Robson Bruniera de Oliveira, Patrícia Brasil

**Affiliations:** 1 Instituto de Pesquisa Clínica Evandro Chagas, Fiocruz, Rio de Janeiro, Rio de Janeiro, Brasil; 2 Serviço de Doenças Infecciosas e Parasitárias, Hospital Universitário Clementino Fraga Filho, Universidade Federal do Rio de Janeiro, Rio de Janeiro, Brasil; 3 Maternidade Municipal Fernando Magalhães, Rio de Janeiro, Rio de Janeiro, Brasil; 4 Secretaria Municipal de Saúde e Defesa Civil do Rio de Janeiro SMSDC-RJ, Rio de Janeiro, Rio de Janeiro, Brasil; Pediatric Dengue Vaccine Initiative, United States of America

## Abstract

**Background:**

Dengue is a reportable disease in Brazil; however, pregnancy has been included in the application form of the Brazilian notification information system only after 2006. To estimate the severity of maternal dengue infection, the available data that were compiled from January 2007 to December 2008 by the official surveillance information system of the city of Rio de Janeiro were reviewed.

**Methods and Principal Findings:**

During the study period, 151,604 cases of suspected dengue infection were reported. Five hundred sixty-one women in their reproductive age (15–49 years) presented with dengue infection; 99 (18.1%) pregnant and 447 (81.9%) non-pregnant women were analyzed. Dengue cases were categorized using the 1997 WHO classification system, and DHF/DSS were considered severe disease. The Mann-Whitney test was used to compare maternal age, according to gestational period, and severity of disease. A chi-square test was utilized to evaluate the differences in the proportion of dengue severity between pregnant and non-pregnant women. Univariate analysis was performed to compare outcome variables (severe dengue and non-severe dengue) and explanatory variables (pregnancy, gestational age and trimester) using the Wald test. A multivariate analysis was performed to assess the independence of statistically significant variables in the univariate analysis. A *p-*value<0.05 was considered statistically significant.

A higher percentage of severe dengue infection among pregnant women was found, *p* = 0.0001. Final analysis demonstrated that pregnant women are 3.4 times more prone to developing severe dengue (OR: 3.38; CI: 2.10–5.42). Mortality among pregnant women was superior to non-pregnant women.

**Conclusion:**

Pregnant women have an increased risk of developing severe dengue infection and dying of dengue.

## Author Summary

Dengue represents a major worldwide public health problem. According to the WHO, up to 50 million dengue infections occur each year. The occurrence of dengue fever and dengue hemorrhagic fever has increased in Brazil, in part due to the simultaneous circulation of DENV-1, DENV-2 and DENV-3. Although a primary infection with one serotype confers a partial or transient immunity against other serotypes, any subsequent infections harbor the risk of increased morbidity/mortality. Several case reports have been published regarding maternal and fetal outcomes from dengue infection, but it is still inconclusive if pregnancy is associated with severity. To estimate the severity of maternal dengue infection, available data that were compiled from 2007 to 2008 by the official surveillance information system of the city of Rio de Janeiro were reviewed. The cases of dengue were analyzed using the 1997 WHO classification. Pregnant women were 3.4 times more prone to developing severe dengue than non-pregnant women. Mortality among pregnant women was superior to non-pregnant women. The increased risk of severe outcomes in pregnant women merits further attention to effective public health and medical interventions that will aid in avoiding morbidity/fatalities within this population.

## Introduction

Since the reintroduction of DENV-1 in 1986 in RJ, dengue has become a major public health problem in Brazil [1]. The occurrence of dengue fever (DF) and dengue hemorrhagic fever (DHF) has increased over the past several years in Brazil, in part due to the rapid spread and simultaneous circulation of the DENV-1, DENV-2, DENV-3 [1]. In 2008, over 600,000 cases of DF and 4,455 cases of DHF were reported in Brazil, with 40% and 42%, respectively, occurring in the state of RJ [2], [3].

A surveillance information system of reportable diseases, SINAN, was implemented in Brazil in the early 1980s [4], and since then, dengue has been a compulsory reportable disease. However, pregnancy was a reportable item on the form only after 2006.

Globally, there are increasing reports of dengue during adulthood, increasing the risk for dengue during pregnancy. In the literature only approximately 400 cases of dengue during pregnancy have been reported, primarily describing the maternal and fetal outcomes [5], [6]. If diseases such as malaria and cholera are more severe during pregnancy, would dengue also be more severe? During the 2007/2008 epidemic in the city of RJ, the highest rate of laboratory-positive dengue samples was among those in the age group under 15 years, followed by those 15–29 years; 99% of all births during this period occurred in mothers aged 15–49 years [7].

To estimate the severity of maternal dengue, the available data provided by SINAN related to the epidemic period of January 1, 2007, through December 31, 2008, in the city of RJ, were reviewed. Laboratory-confirmed dengue cases in reproductive-age women (15–49 years) were included. Mortality and severity of the disease were compared between pregnant and non-pregnant women.

## Methods

### Data source (SINAN form)

A suspected dengue case is routinely reported to SINAN within 24 hours of attendance in a healthcare unit, using a standardized form [8]. When the laboratory results are available, the form is completed by a health staff member who reviews the chart information and adds the final dengue classification, usually after a period of no more than 3 months. Suspected cases are reported from all healthcare facilities in RJ.

The SINAN form includes information on basic demography, laboratory data, hospitalization and outcomes (death or cure). Dengue cases are classified according to the WHO 1997 [9], adapted by the Brazilian Ministry of Health to include the category of dengue with complications [10] for the cases that do not fulfill all three criteria for DHF. Laboratory confirmed cases were considered when either virus isolation, PCR testing, paired IgM or IgG testing or single IgM test was positive.

Pregnancy is categorized in the SINAN form according to trimester: 1^st^ trimester (up to 14 weeks of gestation), 2^nd^ trimester (14–28 weeks), 3^rd^ trimester (after 28 weeks) or unidentified gestational age.

### Study population

#### Eligibility criteria

childbearing-age women with completeness information about pregnancy, dengue classification and laboratory confirmation.

During 2007–2008, of 151,604 suspected dengue cases reported to SINAN in RJ, 76,990 occurred in women. Those with age less than 15 years or over 49 years (n = 17,985) were excluded, resulting in 50,005 suspected dengue cases in reproductive-age women. Laboratory dengue confirmation corresponded to 3,972 cases. Of these, 546 were eligible.

The mean population of reproductive-age women in the city of RJ in the period was 1,700,036: 83,332 pregnant and 1,616,704 non-pregnant women [7].

To estimate dengue-mortality and fatality rates, it was assumed the ratio of 5% [7] pregnancy among childbearing-age women, corresponding to 199 pregnant and 3773 non-pregnant infected women.

Deaths due to dengue occurred in 3 pregnant women and 28 in non-pregnant women.

### Dengue classification

Patients were categorized according to the WHO 1997 classification system as DF, DHF or DSS [10]. Dengue classification of patients (n = 117) categorized in the SINAN form as ‘dengue with complications’ were reviewed. If patients had evidence of plasma leakage they were categorized as having DHF/DSS and thus considered as severe cases. Otherwise, patients were categorized as having DF.

### Statistical analysis

The Mann-Whitney *U* test was used to test the difference between the mean age of pregnant and non-pregnant women and the difference between the mean age of pregnant women by dengue classification (DF and DHF/DSS).

A chi-square test was used to evaluate the differences in the proportion of dengue severity between pregnant and non-pregnant women. A *p*-value of <0.05 was considered significant in all statistical tests.

A univariate analysis was performed using DHF/DSS (dependent variable) and pregnancy, maternal age (as a continuous variable) and trimester (independent variables) using the Wald test. Multiple logistic regression analysis was used to determine whether statistically significant variables were independently associated with dengue severity. Variables with a *p*-value<0.05 in the univariate analysis were included in the multivariate analysis. Finally, the residuals of the fitted model were analyzed. With this modeling, the odds ratio and their respective confidence intervals (95%) were obtained. All statistical analyses of data were performed using *R* software, version 2.11.1.

### Ethics statement

Our study was reviewed and approved by the Ethical Committee of the Municipal Secretary of the City of Rio de Janeiro: Comitê de Ética em Pesquisa da Secretaria Municipal de Saúde e Defesa Civil. Protocolo de pesquisa: 51/08. CAAE: 0122.1.314.000-08 e 0130.1.314.000-08. Inform consent was not obtained because the data were analyzed anonymously.

## Results

The incidence of laboratory confirmed dengue among women in reproductive age was 234/100,000 inhabitants/2y, with similar rates between pregnant (238/100,000) and non-pregnant women (233/100,000). Mortality of dengue was 3,6/100,000 inhabitants/2y among pregnant women and 1,7/100,000 inhabitants/2y among non-pregnant women. Case fatality rate was 7,4 and 1,5% respectively.

Data on 546 eligible reproductive-age women who had confirmed cases of dengue were analyzed: 99 (18.1%) were pregnant and 447 (81.9%) were not (table 1). The mean (± standard deviation) maternal age was significantly different: 26.3±8.5 years in pregnant women compared with 31.5±10.7 years in non-pregnant women (*p*<0.05). No significant difference was observed in the mean age between pregnant women with DHF/DSS (25.5±8.2) and DF (26.9±8.5).

**Table 1:** Mean age, dengue classification, hospitalization and death in pregnant and non-pregnant women of reproductive age.

	Women of reproductive age	
	Pregnant (N = 99)	Non-pregnant (N = 447)
**Age (years)**		
Mean age (± SD)	26.3 (8.5)	31.5 (10.7)
Missing	1 (0.2%)	0
**Dengue criteria (WHO 1997)**		
Dengue fever	53 (53.5%)	364 (77.4%)
DHF	45 (45.5%)	78 (17.5%)
DSS	1 (1.0%)	5 (5.0%)
**Hospitalization**		
Yes	61 (61.6%)	118 (26.4%)
No	3 (3.0%)	4 (0.9%)
Missing response	35 (35.4%)	325 (72.7%)
**Death**		
Yes	3 (3.0%)	5 (1.1%)
No	78 (78.8%)	309 (69.1%)
Missing response	18 (18.2%)	133 (29.8%)

Most cases were classified as DF (n = 417, 76.4%), 123 as DHF (22.5%) and 6 as DSS (1.1%). A higher proportion of pregnant women than non-pregnant women had DHF/DSS (table 1).

Hospitalization information available for 186 (34.1%) patients occurred in 61 (34.1%) pregnant women, and in 118 (65.9%) non- pregnant women. The proportion of severe dengue among hospitalized women was similar: 73.8% and 66.9% for pregnant and non-pregnant women, respectively.

Information on death was available for 395 (72.3%) of the eligible cases: three pregnant and five non-pregnant women died (table 1). Shock syndrome (n = 3) and cavity effusion (n = 2) were associated with deaths. The cause of death was unknown in three patients.

A higher prevalence of DHF/DSS that increased with gestation age was observed (table 2). Pregnant women were 3.4 times more likely to have DHF/DSS, primarily in the last trimester; OR 3.38; CI 2.1–5.42 (table 3).

**Table 2:** Distribution according to the trimester of pregnant women.

	Dengue criteria (WHO 1997)	
	Dengue fever	DHF/DSS
	n (%)	n (%)
**Pregnant**	53 (53.5)	46 (46.5)
First trimester	17 (32,0)	7 (15.2)
Second trimester	11 (20,8)	14 (30,4)
Third trimester	14 (26.4)	23 (50.0)
Trimester unknown	11 (20.8)	2 (4.4)

**Table 3:** Univariate and multivariate analyses.

	DHF/DSS	
	OR (CI)	*p-value*
**Univariate analysis**		
Pregnancy	3.80 (2.40–6.04)	<0.001
Age (15–49 years)	0.97 (0.95–0.98)	<0.001
Trimester		
First trimester	1	
Second trimester	3.10 (0.97–10.6)	0.06
Third trimester	3.98 (1.36–12.65)	0.01
**Multivariate analysis**		
Pregnancy	3.38 (2.1–5.42)	<0.001
Age (15–49 years)	0.97 (0.95–0.99)	0.03
Trimester		
First trimester	1	
Second trimester	3.02 (0.94–10.37)	0.06
Third trimester	3.94 (1.33–12.69)	0.01

## Discussion

This study suggests that dengue during pregnancy can increase maternal mortality, as previously reported [11]. It also suggests that pregnancy is associated with DHF/DSS and that the susceptibility to severe disease increases with pregnancy age.

Severe dengue has been associated with maternal deaths, with fatality rates ranging from 2.9%–22% [5]–[6], [11]–[13]. The maternal dengue fatality in this study was 7.4%. The differences in dengue fatality in pregnant women likely result from differences in the designs and in the heterogeneity of the studies sample sizes. Additionally, it may represent different regional management of dengue in pregnant women.

More than half of pregnant women were hospitalized and it was twice the rate of hospitalization for non-pregnant women, since it was a recommendation of Rio de Janeiro's healthcare authorities to prevent dengue complications in this group. Moreover, the proportion of DHF could still be underestimated as the identification of plasma leakage syndrome through the hemoconcentration or hypoproteinemia may be compromised from the seventh to the 32rd week of gestation, by the physiological increase of intravascular volume of this period [14].

The reasons for the association of DHF/DSS with pregnancy were not assessed in this study. The amount of vascular leakage during early versus late pregnancy may have different effects on the clinical presentation and on the perceived severity level. The higher risk for developing severe disease in the 2^nd^ and 3^rd^ trimesters should be confirmed by prospective studies as the selection bias related to admission because of risk of preterm delivery cannot be excluded.

The non-laboratory confirmed dengue cases were not analyzed to avoid a detection bias, and the confusion of dengue with pregnancy complications, such as HELLP syndrome.

The findings of the study are based on a retrospective review of routinely collected data, with laboratory confirmed dengue, which introduces some limitations such as bias resulted from incomplete data and possible misclassification. Although pregnant women were more likely to be hospitalized for fever and illness in general compared to their non-pregnant counterparts, it would be expected a lower frequency of severity among this group as pregnant women had a preventive hospitalization.

As all the uncompleted data about death were attributed to non-pregnant women, the mortality rate among pregnant women might still be underestimated.

SINAN has also been used in Brazil to conduct studies on dengue [15]. Although citywide surveillance system of information has no specific clinical plasma leakage signs data and may be incomplete, it is a population-base registry from which maternal dengue severity could be inferred by the access to dengue classification. Further longitudinal studies are needed to confirm these findings and to determine on how these two subgroups presents clinically and how their presentations differ.

## Supporting Information

Checklist S1 STROBE Checklist.
(DOC)
